# “What gets measured better gets done better”: The landscape of validation of global maternal and newborn health indicators through key informant interviews

**DOI:** 10.1371/journal.pone.0224746

**Published:** 2019-11-05

**Authors:** Lenka Benova, Ann-Beth Moller, Allisyn C. Moran

**Affiliations:** 1 Faculty of Epidemiology and Population Health, London School of Hygiene and Tropical Medicine, London, United Kingdom; 2 Department of Public Health, Institute of Tropical Medicine, Antwerp, Belgium; 3 UNDP/UNFPA/UNICEF/WHO/World Bank Special Programme of Research, Development and Research Training in Human Reproduction (HRP), Department of Reproductive Health and Research, World Health Organization, Geneva, Switzerland; 4 Department of Maternal, Newborn, Child and Adolescent Health World Health Organization, Geneva, Switzerland; University of South Florida, UNITED STATES

## Abstract

**Background:**

A large number of indicators are currently used to monitor the state of maternal and newborn health, including those capturing dimensions of health system and input, care access and availability, care quality and safety, coverage and outcomes, and impact. Validity of these indicators is a key issue in the process of assessing indicator performance and suitability. This paper aims to understand the meaning of indicator validity in the field of maternal and newborn health, and to identify key recommendations for future research.

**Methods:**

This qualitative study used purposive sampling to identify key informants until thematic saturation was achieved. We interviewed 32 respondents from a variety of backgrounds using semi-structured interviews covering five themes: the meaning of indicator validity, methodological approaches to assessing validity, acceptable levels of indicator validity, gaps in validation research, and recommendations for addressing these gaps. Interview transcripts were analysed data using thematic content approach.

**Results:**

Three conceptually different definitions of indicator validity were described by respondents. They considered indicator validity to encompass meaning and potential to spur action, going beyond diagnostic validity. Indicator validation was seen as an ongoing process of building and synthesising a wide range of evidence rather than a one-size-fits-all cut-off in diagnostic validity tests. Gaps identified included assessing validity of indicators of quality of care and indicators based on facility-level data, as well as expanding studies to a broader range of global settings. The key recommendation was to develop a coordinated approach to summarising and evaluating research on indicator validity, including capacity building in appraising and communicating the available evidence for country-specific needs.

**Conclusion:**

The findings will inform future recommendations around indicator testing and validation.

## Introduction

Recent estimates show that approximately 303,000 maternal deaths, 2.5 million newborn deaths, and 2.6 million stillbirths occur annually worldwide.[1–3] Reducing the substantial burden of preventable maternal and newborn mortality and morbidity is a key priority enshrined in the Sustainable Development Goals (SDGs) [4] and supported by initiatives and strategies such as the Global Strategy for Women's, Children's, and Adolescents' Health [5], Every Newborn Action Plan [6], and Ending Preventable Maternal Mortality.[7] Progress has been made in the past decades, but the pace of change is most likely insufficient to meet the SDG targets for achieving a global average maternal mortality ratio of fewer than 70 maternal deaths per 100,000 live births and a reduction of country-level neonatal mortality to at least as low as 12 per 1,000 live births by 2030.

Several indicators on global, national and sub-national levels are currently used to monitor the state of maternal and newborn health outcomes. Additionally, a range of indicators to track the processes linked with improved health outcomes also exist, including health system inputs, coverage of care, quality of care, as well as equity of access to care. There has been a rapid expansion in the number and range of indicators used by global initiatives to track maternal and newborn programmes.[8, 9] The measurement of maternal and newborn health has a long history of assessing the performance of indicators in capturing their intended meaning, and more broadly, their usefulness in tracking changes that lead to improved maternal and newborn survival. Validity is one consideration in assessing indicator performance and suitability.

This paper is a part of a research portfolio aiming to provide information on how to develop, test and appraise validity of maternal and newborn indicators. It was commissioned by The Mother and Newborn Information for Tracking Outcomes and Results (MoNITOR) technical advisory group which acts as an advisory body to the World Health Organization (WHO) on matters of measurement, metrics and monitoring of maternal and newborn health for the Departments of Maternal, Newborn, Child and Adolescent Health (MCA) and Reproductive Health and Research (RHR).[10] The mandate of MoNITOR is to harmonize and coordinate measurement efforts in maternal and newborn health. Part of this process is sharing research findings and using them to develop standards and norms around measurement of maternal and newborn health.[11] At the meeting of MoNITOR in 2017, a variety of research groups presented about their work on different types of validation research. As a result, MoNITOR commissioned a landscaping review to get a better understanding of the issues within such research, and what validity means and how it can be assessed. However, in the process of conducting this landscaping review, we recognised that even within this key group of people who measure and use maternal and newborn indicators routinely, different understandings of what validity means and how it can be assessed exist.

### Objective

The objective of this study is to understand the meaning of indicator validity in the field of maternal and newborn health, and to highlight key gaps and recommendations for future research based on results of key informant interviews. This paper is a part of a broader situational analysis of validation efforts in maternal and newborn health, with the aim to develop information and a tool-kit on maternal and newborn indicator development, testing and validation.

## Materials and methods

This is a qualitative study using key informant (KI) interviews using semi-structured interviews to understand the scope of validation research within global maternal and newborn health indicators. We followed the consolidated criteria for reporting qualitative research (COREQ)[12] and provide the checklist in S1 Table.

We aimed to answer the following questions, in relation to maternal and newborn indicators:

1. What does the concept of “validity” of indicators mean to various stakeholders?
2. What types of approaches are considered useful in assessing indicator validity?
3. What is an acceptable level of indicator validity?
4. What gaps exist in indicator validation work?
5. What are the recommendations for addressing these gaps?

### Sampling and respondent profiles

We interviewed experts in measurement of maternal and newborn health indicators using purposive sampling until thematic saturation was achieved. First, a list of potential KIs was developed in discussion among the co-authors with input from the MoNITOR co-chairs, and further expanded using snowball methods to encompass experts on the five types of maternal and newborn indicators (health system and input, care access and availability, quality of care and safety, coverage and outcomes, as well as impact). We included experts in both qualitative and quantitative methods to assessing indicator validity. The final sample included 32 respondents, of which 22 were measurement experts based in academic institutions, two were from United Nations agencies, two from implementing agencies, four from organisations funding research and programmes in maternal and newborn health, and two from data collection organisations. Of the potential respondents approached, two suggested an alternative respondent within their organisation due to time constraints or better suitability of expertise. The suggested alternative respondents were interviewed in both cases. All the remaining potential respondents agreed to an interview.

### Data collection methods

Interviews were conducted during face-to-face meetings (six) or by telephone/Skype calls (25) by one author (LB) between December 2017 and November 2018 and ranged between 45 and 90 minutes. During interviews, only the interviewer and the KI were present. One interview (via Skype) included two KIs from one organisation sharing the same job, and the remaining 30 interviews were conducted one-on-one. Each KI was interviewed once. Interviews were conducted in English, guided by a semi-structured interview guide (S1 Material). The interview guide was pre-tested on the first five participants and modified. We decided not to record the interviews and took detailed notes instead. This was done in order to establish rapport with the respondents through a less formal interview setting, and to encourage a frank and open interaction.[13] More structured research topics, such as ours which used a semi-structured interview guide, tend to lend themselves well to manual methods of recording.[14] Detailed notes were taken in shorthand during the interviews, transcribed and expanded immediately following the interview. We aimed to record as much as possible verbatim. Following each interview, further written materials and publications that were referred to by respondents were exchanged through email correspondence with several KIs.

### Data management and analysis

We analysed the interview notes using thematic content approach,[15, 16] reading all interview notes repeatedly to identify relevant themes. We developed a coding framework using a mix of deductive codes generated from the interview guide and inductive codes generated from the initial 12 interviews and expanded after additional 14 interviews. One researcher (LB) performed the coding and thematic content analysis. A continuous consultative approach was employed within the co-author group to reflect on the findings and concepts. After coding all of the interview notes, we identified several emergent themes within each research question, which were further developed using quotes and examples from the published literature referred to by KIs. Further, initial results of this analysis were presented during the MoNITOR technical advisory group meeting in May 2018 for feedback. This manuscript was provided to all KIs in order to provide an opportunity to review whether their responses are potentially identifiable.

## Results

We conducted interviews with 32 key informants. The key themes identified for each research question are presented in Table 1, and elaborated below.

**Table 1:** Overview of research questions and key response themes.

Questions	Key response themes
1. What does the concept of “validity” of indicators mean to various stakeholders?	The meaning of indicator validity means different things to different people, including by different users, languages, and disciplinary backgrounds. Three most common definitions of indicator validity mentioned were: 1. The extent to which an indicator is meaningful to progress. 2. The extent to which the measurement of the indicator corresponds to the construct of interest. 3. Assessment of indicator performance against an objective gold standard (diagnostic validity)
2. What types of approaches are considered useful in assessing indicator validity?	1. A notable change over time in the methods used to assess indicator validity from a focus on methods such as internal consistency, external consistency, reliability, and cognitive interviewing, toward a focus on diagnostic assessment of validity using a gold standard comparison. 2. Recent development and use of new indicators, such as those capturing maternal and newborn health financing, policies, and health system aspects. 3. A shift away from predominantly measuring maternal and newborn care contacts toward incorporating elements of care content and quality. A related phenomenon of a diminished focus on population-based surveys as a source of such data and increasing attention to exploring the quality of routinely collected facility-based data.
3. What is an acceptable level of indicator validity?	There is no objective or recommended cut-off point for a “good” level of diagnostic validity that could single-handedly inform a recommendation to endorse the use of an indicator. Such endorsement relies on additional considerations, such as the intended use of an indicator, quality of data and its source(s), and quality of the gold standard used to evaluate validity.
4. What gaps exist in indicator validation work?	4. Future studies should assess validity of quality of maternal and newborn care indicators, in particular indicators capturing the continuum of care from pregnancy to the postpartum period. Additionally, more work needs to be done on validating indicators derived from facilities and routine data sources. 5. Diagnostic style validity research should continue, and be complemented by rigorous qualitative methods to assess validity (e.g. cognitive interviewing). Studies from a broader range of settings, populations and facility types are needed. 3. Gaps were identified in the communication about indicator validity to a range of country-level stakeholders. Broadly, there is a need to strengthen capacity to collect, analyse, interpret, and use maternal and newborn health indicators.
5. What are the recommendations for addressing these gaps?	1. Better global coordination of validation studies to help avoid duplication of efforts and establish a common language and understanding of indicator validity among the various global and local stakeholders. 2. Development of guidance and criteria for assessment of indicator validity, and prioritising which indicators should be validated first, followed by an “action plan” in cases where an indicator with suboptimal validity is identified. 3. Conducting indicator validation research with a perspective of the needs of low- and middle-income countries. 4. Use the findings of validation studies in order to identify and focus on a smaller number of locally relevant core indicators that lead to action improving maternal and newborn health.

### What does the concept of “validity” mean?

**Fig 1:**
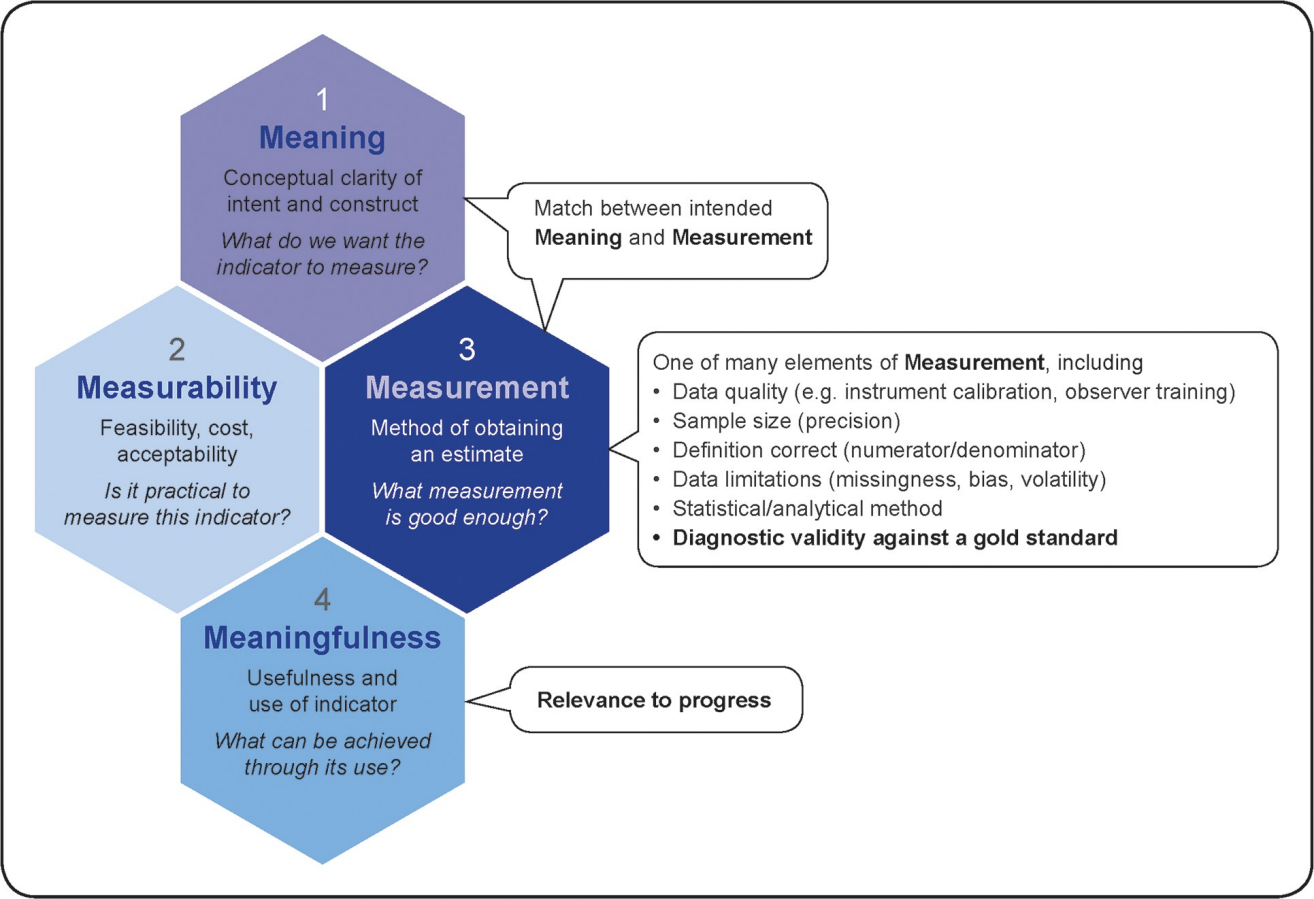
Three most common definitions of indicator validity (text boxes) within a conceptual map of four key indicator measurement concepts (hexagons).

Respondents widely shared the sentiment that indicator validity means different things to different stakeholders, but that it essentially related to how well an indicator measures what it is intended to measure. We constructed a conceptual outline of four concepts related to indicators (meaning, measurability, measurement, and meaningfulness) mentioned by respondents and located the three most commonly mentioned definitions of indicator validity within these concepts (Fig 1). The predominant majority of KIs mentioned more than one of these three definitions of validity, and often all three.

The first, most commonly mentioned and broadest definition of validity, encompassed the entire concept of meaningfulness, or the worthiness of an indicator. Within this viewpoint, KIs noted that indicators must be relevant for the time and place in which they are being used, in order to be agents of progress. To quote a respondent: “an indicator is valid if it leads to positive change”.

The second definition was related to the relationship between the intended meaning an indicator is supposed to capture and the operalization through measurement. In other words, is the technical measurement method appropriate to capture the construct that the indicator is supposed to represent? In the words of one respondent: “Do the measured indicators relate to the facts in the real world? Do they provide a good representation of the real world, a true account of what we want to know about it?” If an indicator is being used as a proxy for a construct that is too difficult or expensive to measure, this should be explicitly recognised in the production and interpretation of such indicator. One example could be an indicator measuring the provision of a routine procedure, such as prevention of postpartum haemorrhage, being used as a proxy for the multidimensional construct of quality of care. KIs commented that in such case, the underlying relationship between the proxy and the concept needs to be clear, in terms of the degree of correlation, extent of linearity of the relationship, and the potential for these relationships to change over time. In this second definition, validity refers to an awareness and continual re-examination of whether indicators measure the construct that they are supposed to be measuring.

The third definition of indicator validity related to diagnostic type validation, or comparison of indicator performance against an objective gold standard. Most KIs mentioned that while this definition of validity is the narrowest, most technical, it is perhaps the most commonly shared understanding of validity among the various stakeholders. However, such acknowledgment was accompanied by a recognition that diagnostic validity is only one of many elements affecting indicator measurement (Fig 1). Several KIs acknowledged that validating against a true gold standard is a positivist perspective, which assumes that the truth is observable and quantitatively measurable. From a qualitative social sciences perspective, the concept of one objective and verifiable truth might not apply, the truth being a social construct and inherently relative and often disputed, and highly dependent on the source of information. The terms used by social scientists for accuracy of indicators were rigour, robustness, trustworthiness of interpretation, which are based on a prolonged engagement, and an in-depth understanding and triangulation of the multiple realities and experiences.[17] Examples of indicators affected by this difference in epistemological perspectives are those capturing women’s experiences (e.g., respectful maternity care, women’s perception of and satisfaction with care, women’s self-reported well-being and morbidity), and those related to the health system (e.g., teamwork, accountability, coordination).

Additionally, several KIs noted that there might be differences in how the concept of indicator validity is understood and applied in various languages. The example cited was its use in French, where validation could mean a process of gaining acceptance for results, or endorsement from stakeholders and experts. In that sense, validity can express the understanding that findings are not true until accepted by the intended users, and thus linking back to its first definition capturing meaningfulness and relevance to progress.

### What types of approaches are useful in assessing indicator validity?

In light of the several conceptual definitions of indicator validity, respondents discussed the various methods used to assess validity, in the perspective of changing needs and opportunities for deriving and using indicators of maternal and newborn health. The three broad themes that emerged include change over time in the mix of methods used to appraise validity, recently developed indicators, and sources of data.

First, many KIs noted that they perceived, in the recent decade, a notable re-orientation from a focus on methods assessing a range of issues related to indicator performance, such as internal consistency, external consistency, reliability, and cognitive interviewing, toward a focus on diagnostic assessment of validity using a gold standard comparison. This shift has affected primarily maternal and newborn health indicators derived from population-level surveys, such as the Demographic and Health Surveys (DHS) or the Multiple Indicator Cluster Surveys (MICS).[18, 19] The inflection point that was mentioned by several KIs was the publication of the Measuring Coverage in Maternal, Newborn and Child Health PLOS Collection in 2013.[20] Since then, many more diagnostic validity studies relevant to indicators derived from population surveys have been conducted,[21–23] and guidance on methodological issues in conducting validation studies of coverage indicators using population-based surveys was published.[24]

A notable exception to the timing of this shift was work on indicators capturing obstetric complications, such as obstetric fistula, haemorrhage, and eclampsia, using women’s recall. KIs familiar with the history of this research noted that the poor diagnostic validity of these indicators was shown over 20 years ago.[25] This was partly due to these conditions being rare, which negatively affected specificity, resulting in large over-estimation of prevalence on a population level.[26, 27] While self-reported reproductive morbidity might capture an element of subjective well-being, the poor validity vis-à-vis gold standard had resulted in such questions being removed from surveys of women. The respondents commenting on this issue saw it as the right decision, which can also inform future action on indicators with poor validity.

From the perspective of the funders supporting diagnostic-style validity assessments of maternal and newborn indicators, the objective of funding such research is to improve measurement relevant to policy and programming, and to support evidence-based decision-making and priority-setting. This perspective was captured well by a respondent who said “what gets measured better gets done better”. Indicators that better reflect the reality can thus lead to more effective action and lead to improvements in quality of care as well as health outcomes of women and newborns.

Overall, KIs mostly welcomed the increased attention to and funding for assessment of indicator validity using comparisons to a gold standard. However, they also noted that continued research on other types of validity, including those related to meaning and meaningfulness, should not be neglected. Additionally, several KIs expressed the need for results of more routine elements of survey question development and testing, such as cognitive interviews, to be published more consistently and made available for other researchers and stakeholders to use. However, additional resources, particularly for population-survey teams within countries and coordinating team, might be required in order to achieve this.

Second, KIs commented on the recent development and use of new indicators types, such as those capturing financing, policies, and health system aspects of the maternal and newborn health field. [8]

For example, should an indicator measuring the existence of a specific policy in a country assess its existence on paper or the strength of its implementation on the ground? If the latter, what are the appropriate data sources to ascertain implementation strength? Indicators of health workforce and financing for maternal and newborn health face additional challenges with inaccurate, sub-optimally disaggregated, or non-existent data sources. One respondent called the process of generating indicators from such sources “archaeology of imperfect data”. Furthermore, the dual purpose of generating these indicators was mentioned by some respondents; “Policy and systems indicators are where there is a tricky balance between advocacy and science”. Some respondents opined that the efforts to understand the accuracy of these indicators are not in fact, in a strict sense, validation studies. Rather, they preferred to use the term verification or triangulation, which might involve understanding of how estimates from models with different assumptions compare.

The third, and perhaps most important shift described by respondents, has resulted from an acknowledgment that indicators of care coverage, such as for example receipt of 4+ antenatal care visits or childbirth in a health facility, have been shown to be relatively poor proxies for maternal and newborn survival.[28–30] The shift has therefore been away from predominantly measuring maternal and newborn care contacts and toward incorporating elements of care content and quality.[31, 32] Studies attempting to adjust indicators of access to care with the receipt of actual evidence-based interventions, or effective coverage, have explored several methods (e.g., individual-record linkage, population-level adjustment) and data sources (e.g., facility surveys, routinely collected HMIS data, women’s recall of receipt of care content). Various methods of calculating effective coverage can also be compared and triangulated, albeit without a gold standard comparison.[33, 34]

The relatively poor performance of key indicators of content of care based on women’s recall, particularly during the intrapartum and early postpartum periods which are crucial to improving survival, has led to a diminished focus on population-based surveys relying on women’s recall. Instead, there has been increasing attention on exploring data quality of routinely collected facility-based data. Such focus was also seen as being in line with the SDGs and contributing to improving data quality and its use by facilities, district, regional and national users. However, KIs mentioned several disadvantages of this approach, for example that increasing reliance on routine data for indicator production causes additional workload on facility staff in low-resource settings, the need for a high level of adaptiveness of routine data sources to incorporate changing clinical guidelines, and the need to understand not just the delivery of routine care components, but also elements needed only by a subset of care users, such as women with complications or small and sick newborns. In this last example, the major difficulty stems from the lack of robust data capturing the *need* for such interventions (the indicator denominator).

### What is an “acceptable” level of indicator validity?

Respondents were asked whether, for quantitative methods of validation assessment, they could define a cut-off point differentiating poor indicator validity from acceptable levels. The predominant reaction was that no indicator is perfect and that some degree of imprecision or inaccuracy can be tolerated. This is in line with the concept of Measurement in Fig 1 –or what is good enough. KIs noted, for example: “it’s not about that it’s perfect, but about how wrong we can afford to be” and “acceptable validity depends on how much imperfection you are willing to put up with and what purpose is the information for”. Some respondents felt that little attention is paid to highlighting and communicating the confidence intervals of indicator estimates to stakeholders who often lack a background in measurement methodology. One respondent noted that: “policy makers are not interested in how things are measured, they only want top line numbers such as maternal mortality or newborn mortality”. Rather than just using point estimates alone (“tweetable numbers”), a better understanding of the levels of imprecision could go a long way toward raising awareness of issues related to accuracy of indicators. This point was raised particularly in relation to situations where multiple estimates of the same indicator might be available (e.g., estimates of maternal mortality ratio), based on different data sources or estimation methods.

There was a strong sense among respondents that there is no objective or recommended cut-off point for a “good” level of validity that could single-handedly inform a recommendation to endorse the use of an indicator. Rather, the assessment of indicator validity was seen on a continuum, a grey scale. One respondent noted that from the statistical perspective, the portraying of indicators as “valid” or “having been validated” is misleading, as statistical methods can only determine whether a hypothesis is incorrect, rather than the opposite. Beyond the quantitative measures of diagnostic validity, such as sensitivity, specificity, and inflation factor, respondents mentioned several additional perspectives to take into consideration when assessing the level of indicator validity.

Three key considerations mentioned by respondents related predominantly to concepts 2 and 3 (measurability and measurement) on Fig 1. Firstly, the acceptable level of accuracy and validity depends on the intended use of an indicator. For example, is the indicator used to assess programme performance or the national situation? Does it inform decisions on a country-level or used for comparisons across countries? Is it used to monitor progress over time, and if so, how sensitive is the indicator to expected changes? Respondents also noted that some indicators with “poor” validity might be worth collecting, for example to improve data quality and use in the future.

The second consideration related to the extent of variability in an indicator’s validity performance, over time and across various settings (countries and regions) and levels of disaggregation (e.g., regional, socio-economic status, facility levels). What is the validity of indicators capturing highly volatile situations, such as availability of essential supplies or running water? How do various data sources compare in terms of data quality in general and validity in particular? What is the feasibility and cost of producing indicator estimates? Might a slightly less valid, but more economical, indicator be an acceptable alternative?

Third, KIs stressed the importance of considering the quality of the gold-standard measure itself. This issue is particularly relevant to indicators capturing the coverage of targeted interventions, such as newborn resuscitation or treatment for neonatal sepsis. Issues of validation are even more acute for the denominators of such indicators—the women and newborns who needed the intervention—but which are challenging to obtain, due to issues of subjectivity and potential for data manipulation when routine facility data is used. The quality and appropriate design of validation studies depends on having a sufficient sample size, an issue for studies validating indicators of interventions with high coverage (e.g., childbirth being attended by a skilled birth attendant within facility), leading to inability to assess specificity. Potential for selection bias within validations studies should also be carefully assessed. In this regard, respondents commented on issues related to facility-based studies of indicator validity. They noted that the sub-population of women and newborns who did not receive facility-based care is excluded from such samples and hence the validity of the population-level indicator remains unknown. Similarly, there were concerns that validation results conducted in a small number of facilities are not generalizable across the range of facility types (e.g., public, private and voluntary sectors) and levels (primary, secondary, and tertiary).

Several respondents commented on levels of validity related to indicators derived from population-level surveys. Specifically, they noted that the existence of these surveys and indicators for several decades might lead to a perception of these surveys being “validated tools” or “valid data sources”. While these questions might have been tested using various methods, a substantial proportion of the questions contained on these surveys have not been assessed for diagnostic validity. This potential misperception is also the reason to advocate for prompt removal of questions that are found to perform poorly (as on the example of obstetric complications), otherwise the presence of such questions on surveys, and resulting indicators, might be interpreted as an endorsement of their validity. Some respondents noted that because these nationally representative surveys are used for cross-country comparisons, the expectation is that the indicators derived from them should meet a more rigorous standard of validity.

### What gaps exist in indicator validation for maternal and newborn health?

Several gaps and potential opportunities were identified by the KIs, and these have been organised into three key themes–indicators for which to prioritise conducting validation studies, use of a broader range of methods and settings, and communication of validation results.

First, consistent with the increased attention on care quality; nearly all respondents noted the need to conduct further studies assessing validity of indicators capturing quality of maternal and newborn care. Currently used indicators, such as newborns receiving essential newborn care and early initiation of breastfeeding, were seen as measuring care content or comprehensiveness, which is only one dimension of a much broader construct of quality of care. Respondents advocated for further validation work focusing on indicators of patient-centred care, such as satisfaction and respectful care, while acknowledging that diagnostic validity assessments against a gold-standard are not suitable to understand the performance of such indicators. However, the compilation and use of such indicators can contribute to valuing of women’s experiences, or, as one responded noted, “we need to listen to what women are saying”.[35]

Furthermore, respondents advocated for making progress in developing and validating more sophisticated indicators capturing the continuum of care from pregnancy to the postpartum period, for both the woman and the newborn. Such indicators might have the scope for local adaptation based on differing care guidelines and practices, but would require heavy reliance on observations or medical records/facility registers to obtain data on gold-standard. The need to assess validity of indicators capturing abortion care was also mentioned, as was the inclusion of care for pregnancies without a live birth outcome in indicator definitions. A gap was perceived in the understanding of validity of indicators capturing maternal and newborn morbidity, including non-communicable diseases, and of health and well-being during and beyond women’s reproductive lifespan.

In terms of health systems indicators, respondents noted that more work needs to be done on validating indicators derived from facility and routine data systems. Research on indicators and dimensions that can be measured comparably across countries should be prioritised. In a more general sense, KIs raised the question of usefulness of “static” health system indicators, and the need to develop and validate indicators which reflect the dynamic, complex and non-linear character of health systems. It would be important to explore relationships between various indicators of health system inputs, and their correlation with maternal and newborn health outcomes, as a secondary means of assessing validity. However, this would need to be accompanied by an in-depth understanding how measuring and using specific indicators might perversely influence incentives, and the feedback loop leading to production of data and indicators.

Second, while respondents agreed that diagnostic style validity research should continue, they also advocated for more qualitative approaches to validity to help explore, for example, how survey questions are understood by survey enumerators and by women. Respondents also highlighted a gap in the coverage of a broader range of countries. There was an acknowledgment that some validation research, because of its expense, takes place within other projects, and might be restricted to countries with higher levels of research capacity. Respondents noted that much of recently published diagnostic validity research was conducted in sub-Saharan Africa and Asia, with gaps in evidence from the Middle East, Eastern Europe, and Latin America. Knowledge gaps in our understanding of levels of maternal and newborn health indicator validity in conflict-affected countries and nomadic/displaced populations were also mentioned. Additionally, respondents saw a gap in the understanding of validity of women’s recall of care received in lower-level health facilities and those receiving home-based care.

Third, respondents mentioned gaps in the communication of information about indicator validity, particularly within LMICs, and its link to the need to strengthen capacity to collect, analyse, interpret, and use maternal and newborn health indicators to spur and sustain improvement. There is a need to improve understanding of how indicators are used and useful within countries. One suggestion was to more inclusively engage researchers and policy-makers from LMICs within global discussions on maternal and newborn health indicator assessment, selection, and suitability for their settings. Within such supportive environment, validation studies could directly help countries select a subset of indicators useful for their situation, and experiences of this process could be shared across countries. Last, some respondents acknowledged that validation studies are expensive, and not the first priority for funders in the maternal and newborn health space. Bill and Melinda Gates Foundation, Children's Investment Fund Foundation and USAID were seen as the most important funders of current research to assess maternal and newborn health validity. Hence, respondents recommended that gaps in validation research, and the advantages that accrue from improving measurement, should be more effectively communicated toward other potential funders.

### What are the recommendations for addressing the gaps in validation?

Four key themes on recommendations for work on validation of maternal and newborn health indicators emerged from the interviews.

First, most respondents suggested that a better global coordination of validation studies could help avoid duplication of efforts and establish a common language and understanding of “indicator validity” among the various global and local stakeholders. A centralised approach toward identifying, synthesising and disseminating results of published and future studies, including from the grey literature, would be appreciated. Respondents suggested that such synthesis be made in an accessible way to various stakeholders, and be explicit about the key construct(s) an indicator is intended to measure, and where it might fall short of such goal.

Second, respondents expressed the need to develop guidance and criteria for assessment of indicator validity, and prioritising which indicators should be validated first, followed by an “action plan” in cases where an indicator with suboptimal validity is identified. This would include discontinuing the use of an indicator and exploring other methods or data sources to better capture the intended construct. In regard to actions taken on the basis of validation studies, there were two opposing views from respondents. Some found that the response has been appropriate and sufficient, mentioning examples of when survey questions or indicators were revised to reflect validity findings (e.g., DHS questions on immediate skin-to-skin contact [36, 37]). However, others felt that the response to studies showing poor validity of some maternal and newborn indicators, predominantly those based on population-level surveys, was reluctant and slow. This was particularly the case for indicators that have been in use over a long period of time. Yet another perspective was that stakeholders needed to be cognizant that it takes time for a questionnaire to be developed, collected, and data to be made available. Indicators derived from surveys therefore need to be fairly constant over time, otherwise the available estimates are perpetually outdated, not fit for purpose, and not comparable over time. The need for close collaboration with the population survey teams (e.g., MICS and DHS) was emphasized in order to benefit from their expertise in the design and implementation of validation studies, and to promote buy-in of results.

Third, the strongest theme within respondents’ recommendations was to work on indicator validation with a perspective of the needs of LMICs. One respondent noted that “Global donors are obsessed with indicators and cross-country comparisons, but national policymakers don’t care about cross-country comparisons or comparability. Many respondents noted that such focus on cross-country comparability of indicators is unhelpful in tracking and improving maternal and newborn health on a country level. Rather, they suggested to work on improving the understanding of country-level needs and processes, and engaging national stakeholders in discussions on indicator validation. Further, some respondents believed LMIC policy-makers were not particularly interested in technical aspects of validation and measurement of indicators. Rather, the need is for context-appropriate, sub-nationally disaggregated, and regularly updated indicators. A handful of respondents also mentioned the discrepancy between the high number and variety of indicators expected of LMICs when compared to high-income countries (HICs). They suggested that the field would benefit from an improved understanding of the indicators that have been collected and monitored in HICs and how they were used to achieve progress in maternal and newborn health in such settings.

The final recommendation was to use the findings of validation studies in order to identify and focus on a smaller number of locally relevant core indicators that lead to action. Approaches to indicator validity taken by the HIV/AIDS community were cited by several KIs to highlight that progress has been partly attributed to the use of a small number of globally-agreed indicators which are aligned with HMIS data production, and linked to clear goals (i.e., “90-90-90”).[38] While acknowledging that maternal and newborn health indicators face more difficult issues of validity (e.g., inability to rely on biomarkers), respondents stressed that the field should not be paralysed by concerns over indicator validity. Instead, they recommended that the interest in, and sometimes scepticism about, indicator validity in maternal and newborn health is not to the detriment of this field of global heath. Rather, identifying and using the best possible indicators can be very powerful in order to advocate for investments into maternal and newborn health.

Prioritising the use of fewer indicators should result in improvement in data quality, while reducing the cost of data collection and indicator production. Respondents noted that the “data architecture” for collection of routine facility data and civil registration and vital statistics is of suboptimal quality in many LMICs. Thus, while new technology might improve the ability to measure some aspects of maternal and newborn health, such as quality of care, a reduction in the number of indicators measured can provide an opportunity to focus on improving the system of data production. Several respondents suggested that a global coordination effort for validation of maternal and newborn health indicators might be able to recommend a different list of core indicators for countries of various levels of development and health systems. Respondents had a powerful sense that efforts on assessing indicator validity should be directly linked to action—to quote—“an indicator is valid if the expenditure on action based on the indicator is justified–i.e., that it is consistent with the values and goals of the health system at that point in time.”

## Discussion

This study addresses a gap in the existing literature through synthesising the various understanding of validity and validation research. A recent scoping review identified 140 indicators linked to maternal and newborn health topics across the continuum of service provision.[8] As efforts to meet the SDGs accelerate ahead of the 2030 deadline, our results directly link with a global agenda to strengthen measurement in reproductive, maternal, newborn, child and adolescent health and nutrition (RMNCAH-N) in general and in maternal and newborn health in particular.[39] One key aspect of this agenda is generating a core set of *validated* indicators.

We used qualitative methods to explore the meaning and importance of indicator validity used for monitoring of maternal and newborn health globally. Many of the findings, summarised in S1 Box, are not limited to maternal and newborn health, but relevant to indicators from other areas in global health. The main findings show that respondents consider indicator validity as tightly entwined with other issues in the production and use of indicators globally and nationally, including indicator development and testing, improvement of data quality, and appropriate communication of resulting indicator estimates. There was a strong sense among KIs that “validity” goes beyond merely technical, diagnostic aspects–and spans across dimensions of meaning, usefulness and action to improve the health of women and newborns. We identified a variety of understanding and usage of the term “validity” across disciplines, languages, indicator types, data sources, and stakeholders.

Respondents viewed indicator validation as an ongoing process of building and synthesising evidence. They advocated for more research assessing validity in a broader range of settings. There was considerable scepticism about the possibility of identifying one standard definition of an acceptable diagnostic validity level. Instead, KIs recommended that decisions to support the use of indicators should assess a broad range of evidence, including cognitive interviews, field testing, acceptability, accuracy, and validity—the type of evidence that requires continued funding in order to be generated. Despite the acknowledged complexities in developing guidance on assessing indicator validity, most respondents thought that a coordinated approach to summarising and evaluating research on validity would be extremely useful. Some guidance on evaluation of indicator performance is already available [24, 40] and future coordination efforts can be informed by respondents’ suggestion that while indicators with poor validity should not be used, a good diagnostic validity does not mean an indicator is necessarily used and useful in leading to action and improvement.

Some key maternal health indicators, to a much greater extent than newborn indicators, have a decades-long history of measurement and monitoring. This long track record and insistence on comparability over time and across countries might make decisions about discontinuation of indicators with poor validity performance more difficult and hinder development and use of more useful, valid indicators. Considering the large number of available indicators, respondents communicated a sense that the number of indicators tracked should be reduced, leading to prioritisation of core indicators that can help achieve gains in maternal and newborn survival. It is imperative that such efforts be conducted with meaningful participation from LMIC stakeholders, and take account of specific country needs. Respondents suggested that HICs’ experience with the collection, calculation and monitoring of maternal and newborn health indicators might be informative in this process.

This study used qualitative methods allowing to gather a detailed and varied perspective on indicator validity. The number of KIs was not determined at the onset of the study; rather, it was reached at the point of thematic saturation. Respondents included collaborators on many recently published studies assessing maternal and newborn indicator validity, as well as experts with historical perspectives on validation. We interviewed a wide range of stakeholders involved in validation of the different types of indicators. This is an important consideration as many respondents focused very closely on the type of indicators or data source they work with, rarely mentioning issues of validity for the whole spectrum of maternal and newborn indicators (input, process, coverage, outcome). However, we acknowledge that our sample included KIs working predominantly on the global level, rather than national stakeholders from LMICs. The preliminary findings of this study were presented to various audiences which included representatives of LMICs; their input into the interview guide and interpretations of results was taken into consideration.

## Conclusion

The findings from key informant interviews on validation of maternal and newborn health indicators presented in this paper provide timely and diverse perspectives on the measurement challenges in this field. They also constitute an important step in the process of developing guidance on the measurement, appraisal and use of maternal and newborn indicator validation.

## Supporting information

S1 Table Consolidated criteria for reporting qualitative studies (COREQ): 32-item checklist. (DOCX)

S1 Material Interview guide. (DOCX)

S1 Box Key messages. (DOCX)
