# Supplementary material for: “What gets measured better gets done better”: The landscape of validation of global maternal and newborn health indicators through key informant interviews
Source: PLoS One. 2019 Nov 5;14(11):e0224746. doi: 10.1371/journal.pone.0224746 (PMC6830807; doi:10.1371/journal.pone.0224746)
Supplement: S1 Table — (DOCX) [file pone.0224746.s001.docx]

**S1 Table.**

| **No. Item** | **Guide questions/description** | **Reported on Page # or Section** |
| --- | --- | --- |
| **Domain 1: Research team and reﬂexivity** | | |
| *Personal Characteristics* |  |  |
| 1. Interviewer/facilitator | Which author(s) conducted the  interview or focus group? | Lenka Benova |
| 2. Credentials | What were the researchers’ credentials? E.g. PhD, MD | LB: MA, MSc, PhD  A-BM: RN, RD, MPH  ACM: MHS, PhD |
| 3. Occupation | What was their occupation at the time of the study? | All authors are researchers in the field of reproductive, maternal and newborn health. |
| 4. Gender | Was the researcher male or female? | All researchers are female. |
| 5. Experience and training | What experience or training did the researchers have? | The researchers have training in qualitative and quantitative research methods in epidemiology and public health, and years of experience with programmes and research on maternal and newborn health in LMICs. |
| *Relationship with participants* |  |  |
| 6. Relationship established | Was a relationship established prior to study commencement? | One author conducted the interviews (LB). She was acquainted professionally with some of the KIs through previous collaborations, and met other KIs for the first time during these interviews. |
| 7. Participant knowledge of the interviewer | What did the participants know about the researcher? e.g. personal goals, reasons for doing the research | Participant were informed about the research aims by email and upon agreeing to participate had further opportunity to ask questions before, during, or following the interview. |
| 8. Interviewer characteristics | What characteristics were reported about the interviewer/facilitator? e.g. Bias, assumptions, reasons and interests in the research topic | LB provided the KIs with an introduction about the research and her role in the research as a grant recipient from the WHO to the LSHTM, where she was a staff member at the time of the interviews. She also briefly explained her background in quantitative methods. |
| **Domain 2: study design** | | |
| *Theoretical framework* |  |  |
| 9. Methodological orientation and Theory | What methodological orientation was stated to underpin the study? e.g. grounded theory, discourse analysis, ethnography, phenomenology, content analysis | Methods |
| *Participant selection* |  |  |
| 10. Sampling | How were participants selected? e.g. purposive, convenience, consecutive, snowball | Methods |
| 11. Method of approach | How were participants approached? e.g. face-to-face, telephone, mail, email | Methods |
| 12. Sample size | How many participants were in the study? | Methods |
| 13. Non-participation | How many people refused to participate or dropped out? Reasons? | Methods |
| *Setting* |  |  |
| 14. Setting of data collection | Where was the data collected? e.g. home, clinic, workplace | Methods |
| 15. Presence of non-participants | Was anyone else present besides the participants and researchers? | Methods |
| 16. Description of sample | What are the important characteristics of the sample? e.g. demographic data, date | Methods |
| *Data collection* |  |  |
| 17. Interview guide | Were questions, prompts, guides provided by the authors? Was it pilot tested? | Methods and Supplementary material 2 |
| 18. Repeat interviews | Were repeat inter views carried out? If yes, how many? | Methods |
| 19. Audio/visual recording | Did the research use audio or visual recording to collect the data? | Methods |
| 20. Field notes | Were ﬁeld notes made during and/or after the inter view or focus group? | Methods |
| 21. Duration | What was the duration of the inter views or focus group? | Methods |
| 22. Data saturation | Was data saturation discussed? | Methods |
| 23. Transcripts returned | Were transcripts returned to participants for comment and/or correction? | Methods |
| **Domain 3: analysis and ﬁndings** | | |
| *Data analysis* |  |  |
| 24. Number of data coders | How many data coders coded the data? | Methods |
| 25. Description of the coding tree | Did authors provide a description of the coding tree? | No |
| 26. Derivation of themes | Were themes identiﬁed in advance or derived from the data? | Methods |
| 27. Software | What software, if applicable, was used to manage the data? | Methods |
| 28. Participant checking | Did participants provide feedback on the ﬁndings? | Methods |
| *Reporting* |  |  |
| 29. Quotations presented | Were participant quotations presented to illustrate the themes/ﬁndings? | Results |
| 30. Data and ﬁndings consistent | Was there consistency between the data presented and the ﬁndings? | Discussion |
| 31. Clarity of major themes | Were major themes clearly presented in the ﬁndings? | Results |
| 32. Clarity of minor themes | Is there a description of diverse cases or discussion of minor themes? | Results |

Based on: Tong A, Sainsbury P, Craig J. Consolidated criteria for reporting qualitative research (COREQ): a 32-item checklist for interviews and focus groups. *International Journal for Quality in Health Care*. 2007. Volume 19, Number 6: pp. 349 – 357.
