# Supplementary material for: “What gets measured better gets done better”: The landscape of validation of global maternal and newborn health indicators through key informant interviews
Source: PLoS One. 2019 Nov 5;14(11):e0224746. doi: 10.1371/journal.pone.0224746 (PMC6830807; doi:10.1371/journal.pone.0224746)
Supplement: S1 Material — (DOCX) [file pone.0224746.s002.docx]

**S1 Material.**

*Part 1: General – understanding key themes and issues in indicator validation*

Introductions

Describe the purpose of interview

Provide scope of indicators that are of interest within **maternal/newborn health** (current/aspirational; survey/facility/ DHIS/policy; maternal/newborn; coverage/content).

Questions

1. Extent of engagement in validation work and landscape of validation work

- What kind of work relevant to validation of maternal/newborn indicators is your organisation currently doing, if any?
- Are you conducting, planning to conduct or have you conducted any validation studies? (if yes – also administer Part 2)
- Have you commissioned any validation work or thought about using data collected for other purposes to validate any indicators?
- Who are other stakeholders (organisations, researchers) conducting validation that you know?
- What do you think is the motivation behind work on validation?

1. What does validation mean?

- Is validation of indicators relevant to your work? If so, how?
- In your view, what types of research can be considered “validation”?
- What do you understand by the concept of “validity”?
- What is considered to be good versus poor validity?
- What is the perceived value of doing validation and where does funding for validation come from?
- What is the generalisability of validation findings (over time, across contexts and settings, data sources) How long is an indicator validated for (i.e., what is the need for re-validation)?

1. Recommendations of future validation work and uptake of findings

- What is the global and local appetite for validation and its results? Are validation results “accepted” and taken up?
- How are national governments and regional-level actors participating on validation work?
- Do you perceive a gap in which indicators are/aren’t being validated and why?
- What other gaps in work on indicator validation do you see?

Wrap up

Is there anything else you’d like to share on this topic?

May I get in touch with you with any questions?

Thank you for your time. Agree on follow up if any materials were to be sent.

*Part 2: For data collection on validation work* (interviews or from written materials, such as reports, published literature and pre-publication drafts)

Information to be collected for each project, from the respondent or by email (study protocols, study findings, etc; published and unpublished)

Country(ies)

Setting - implementation, M&E, stand-alone work/opportunistic, facilities, households

Funding source

Timeline and expected results

Type of validation applied – methodology (sample size, gold standard, statistical indicators used)

What is considered to be good v poor validity and based on what criteria - statistical performance, variability?

Can you share the study protocol or any other materials to help understand the details of the work being conducted, including data collection and data analysis strategies?

Additionally, for projects that have results:

Can you share results with me (unpublished findings, study protocols, check that I identified all published materials)

What variability in validity has been found? What are the reasons for this variability?

How were results disseminated and received?

Anything you would have done differently?
