# Supplementary material for: “What gets measured better gets done better”: The landscape of validation of global maternal and newborn health indicators through key informant interviews
Source: PLoS One. 2019 Nov 5;14(11):e0224746. doi: 10.1371/journal.pone.0224746 (PMC6830807; doi:10.1371/journal.pone.0224746)
Supplement: S1 Box — (DOCX) [file pone.0224746.s003.docx]

**S1 Box.**

#### What is already known?

- A recent scoping review identified 140 indicators linked to maternal and newborn health topics across the continuum of service provision.
- Understanding indicator validity is a critical issue that will allow further development and prioritisation of maternal and newborn health indicators for monitoring progress and priority intervention areas.

#### What are the new findings?

- This research identified three unique types of understanding of the concept of “indicator validity”.
- Important shifts in validation research have taken place in recent years – toward diagnostic-style validation methods, toward exploring the use of routinely collected health system data for indicator construction, and toward developing and validating indicators of care quality.

#### What do the findings imply?

- There is a high priority for more coordination and collaboration in designing, conducting, synthesising and acting on validation studies, and including low- and middle-income stakeholders in these processes.
- More resources toward validation research are needed, in particular to expand the range of country contexts studied and to apply a broader variety of methods to assessing accuracy and validity.
